# A Conceptual Model for Strengthening Family Capabilities Through a Process of Care

**DOI:** 10.3390/ijerph22071150

**Published:** 2025-07-20

**Authors:** James Reid, Chanté Johannes, Shenaaz Wareley, Collen M. Ngadhi, Avukonke Nginase, Nicolette V. Roman

**Affiliations:** 1 School of Education, University of Huddersfield, Huddersfield HD1 3DH, UK; 2 Centre for Interdisciplinary Studies of Children, Families, and Society, University of the Western Cape, Cape Town 7535, South Africa; chjohannes@uwc.ac.za (C.J.); 3814572@myuwc.ac.za (S.W.); nmcollen@gmail.com (C.M.N.); 4024125@myuwc.ac.za (A.N.); nroman@uwc.ac.za (N.V.R.)

**Keywords:** ethics of care, family, family capabilities, qualitative, South Africa

## Abstract

Family capabilities shape the well-being of individuals and families, particularly in diverse sociocultural contexts. However, existing frameworks often fail to capture the complexities of family dynamics, particularly in South Africa. This limitation is addressed in this study by developing a new conceptual model, through an ethic of care lens, to understand family. A Human-Centered Design strategy is employed, utilizing workshops to gather and deductively interpret data. A sample of 53 participants is recruited from two communities in South Africa. The Listening Guide approach is used to analyze the data, alongside thematic analysis, to identify overarching themes. The findings reveal tangible needs (material and physical requirements), as well as intangible needs (emotional and psychological support). The relation between these needs and harms highlights how care is shaped in families. While families endeavor to meet intangible needs, many fail to communicate these intangible needs, which also leads to harm. This is exacerbated when the primary caregiver within the family, the ‘glue’, has needs for care that are not met. In addition, the findings reveal the interrelatedness of care in family dynamics among family members, and help to explore the boundaries, capacities, and capabilities of what families do and how they care.

## 1. Introduction

In this paper, the authors highlight the development of a new conceptual model to understand family capabilities through a South African lens. The South Africa National Development Plan (NDP) 2030 [1] assumes that a reciprocal and bi-directional relationship exists between families and the systems they encounter, which facilitates social change and transformation. The approach in the NDP draws on the Capabilities Approach (CA) developed by Sen [2,3], as well as the human xapabilities approach developed by Nussbaum [4], and focuses on the key capabilities that people need to live the life that they desire. However, currently, no understanding or model of the relationship between families and capabilities exists, nor between families and capabilities policy [5,6]. In this context, the authors aim to make an original contribution to explicate the choices available to families, as well as what they ‘do’ to achieve, or not achieve, well-being and the lives they desire.

The discussion starts with the consideration and definition of family in the South African context. The authors briefly introduce the concept of family capabilities, emphasizing an approach that requires the exploration of what families ‘do’. Subsequently, they explore the themes developed and offer a conceptual framework for the understanding of families, as well as their everyday being and doings.

### 1.1. The Family, in the South African Context

Defining ‘family’ is a complex endeavor that varies across disciplines, as well as social, economic, legal, and cultural contexts. In addition, it includes government, regulatory bodies, agencies, researchers, academics, and communities, as well as families themselves [7]. These diverse agents complicate any widely accepted or universal definition of family [8]. However, family researchers have endeavored to conceptualize the concept of family through various dimensions, including its structure, interdependence, interactions, and relationships [5,8]. The family has been defined as a fundamental social institution that is essential to the functioning of any society and, consequently, serves as a potential foundation for the creation and maintenance of social cohesion [9]. In the African context, families are characterized as extensive networks in which members provide mutual support, socialize children, and care for vulnerable individuals within the family, as well as the broader community [10]. The Revised White Paper on Families in South Africa [11] defines family as a “societal group that is related by blood (kinship), adoption, foster care or the ties of marriage (civil, customary or religious), civil union or cohabitation, and goes beyond a particular physical residence” (p. 10). This is a pragmatic definition, as the White Paper also recognizes the near impossibility of a definition to cover all types, contexts, experiences, and situations. In addition, the White Paper acknowledges the role of historical, enduring social inequality and discrimination that has impacted families, as well as family cohesion [11]. However, when defining ‘family’ in the South African context, it is important to consider the types of families that are present in society, so as to capture the dynamics and fluidity of the term [12,13]. For example, same-sex marriages are legalized and recognized in the country [11]; single-parent families, predominantly headed by females [11], with absent fathers being the most popular [14], are innumerous, while nuclear families are among the least common family [11]. Additionally, the families in the country are characterized by skip-generation households, in which children are reared by grandparents [11]. According to the General Household Survey 2023 [15], approximately 70% of primary caregivers are grandmothers; consequently, this emphasizes the significance of extended family structures and support. Twice as many (28%) extended family households exist in rural areas as opposed to urban areas (14%) [15]. In addition, child-headed households represent a significant and growing family structure [16].

Therefore, for this paper, ‘family’ is defined beyond the boundaries of blood and union, as families can be defined by the emotional, social, and legal bonds between individuals [17]. The term includes adoptive families, stepfamilies, and chosen families formed by close friends who share mutual care and responsibility. For example, Pearce et al. [18] emphasize the changing nature of families and, in particular, highlight the complexities of attempting to examine family structures while conducting research. Similarly, expanding on the evolving concept of family definitions, Kim and Feyissa [19], as well as Thomas and Smith-Morris [20], explore chosen families as non-biological bonds rooted in emotional trust and safety. They draw a comparison between biological families and chosen families. Chosen families can emerge when the biological family may have ostracized the family member, when an immigrant has left their biological family behind, or when the root family is in crisis [21,22]. These are scenarios where non-traditional members fulfill familial responsibilities. Kim and Feyissa [19] state the following:

“Biological family is sometimes associated with words that instigate fear, danger, and insecurity, while the concept of chosen family is associated with words like trusting, like-minded, understanding, welcoming, loving, committed, etc.”

(p. 1)

The conventional family, therefore, is disrupted [8], and a redefinition of the family concept may be required. The redefinition of families implies that the family constitutes a dynamic, adaptive network of individuals incorporating various structures, such as chosen families, non-kin caregiving relationships, and flexible arrangements, including cohabiting partners and multi-partner fertility configurations, which surpass conventional biological, legal, or marital definitions. Consequently, this redefinition may require a different response to meet the needs of different families, underscoring the need to examine how such arrangements enable, or constrain, access to critical resources (for example, emotional support and financial stability) and agency, which aligns with the focus of the human capability approach, to foster conditions for collective flourishing.

### 1.2. Developing an Understanding of Family Capabilities

Theoretically, the human capability approach is grounded in the works of Amartya Sen and Martha Nussbaum [2,3,4,23], emphasizing the conditions necessary for individuals to lead fulfilling lives and framing capabilities as fundamental to human development, as well as well-being [24]. In response to various approaches to quality of life that highlight welfare, or resources, as elements of primary consideration, Sen [25] argues that an appropriate evaluative measure that facilitates an understanding of quality of life is an assessment of the ‘doings and beings’ which are available to an individual, both as freedoms and as achieved states of being. In addition, he frames this assessment in terms of functionings and capabilities, with functionings referring to achieved states of being (namely, being nourished and having bodily integrity) and capabilities being the substantial opportunities and freedoms available to reach these achieved states of being [25,26].

To avoid emphasizing material factors for well-being (resources, income, and wealth), it is necessary to consider how an individual converts resources into what they can do and be (namely, capabilities). This involves personal characteristics, social characteristics, and environmental characteristics [25,27] that enable, or constrain, the conversion of resources, as well as internal abilities and potentials, into capabilities and functionings. The Capabilities Approach (CA), therefore, emphasizes the substantive opportunities available to individuals, enabling them to choose what to be and do, for example, to be healthy and to participate with others in social and community life [28]. Capabilities reflect the range of functionings that are accessible and available to an individual [29]. They reflect what an individual (or family) has access to in terms of internal competencies and abilities, as well as external resources, support, and enabling structures.

Sen’s work is further developed by Nussbaum [23], who posits fundamental human capabilities that include life, bodily health, bodily integrity, the development and expression of the senses, imagination and thought, emotional health, practical reason, affiliation, relationships with other species, play, and control over one’s environment. Additionally, there is a developing body of work that posits the potential of perceiving capabilities as features of collectives (such as the family), not only of individuals [30]. The intention of focusing on the collective, or group, is to show the strength of shifting individuals towards flourishing, or well-being [28].

However, before discussing collective capabilities, a discussion of groups should be conducted first. Social Identity Theory (SIT) [29] provides an understanding of how groups are formed, how they function, and how they are understood. More specifically, it provides a perspective on how and why an individual is drawn to a particular group, as well as how the individual assimilates and adapts to the group [30,31]. According to [32], as well as Bryant and Bainbridge [33], members of social groups obtain guidance, structure, direction, and meaning from these social groups. Therefore, in the context of social groups, social identity is a way in which individuals perceive themselves in relation to the groups they are associated with or to which they belong [34]. This suggests experiencing a sense of belonging, or relatedness, to a group, which could include sharing the same history, culture, values, and beliefs, as well as knowing how others in society perceive that group [35]. Social identity could have a significant effect on an individual’s sense of self, as well as their behavior and attitude towards members of other groups. It is a complicated and dynamic process that is susceptible to various factors, including personal experiences, social norms, and cultural expectations [36,37].

In the same way that social identity unpacks the complexities of groups, collective capabilities provide another aspect of groups [38]. Collective capabilities, as an aspect of capability theory, are a way of observing how people grow and thrive within a group [38]. They are a way in which groups, or communities, can work together to reach shared goals and improve everyone’s lives, holistically [31,35]. Collective capabilities acknowledge that people are part of social structures, and what their community accomplishes as a whole shapes their chances and successes [35,39]. For example, in a neighborhood where unemployment and poverty are prevalent, residents could form a group that strives to improve economic chances for everyone. They could combine their money, skills, and knowledge to form a cooperative business model, such as a small-scale community manufacturing project. By working together, they can create jobs, earn money, and deal with economic problems that affect their well-being as a collective. Robeyns [40], as well as Adabanya et al. [41], assert that a group, or collective, must participate in joint action in order to reach a capacity that the group’s members value. Consequently, a group’s collective capabilities are used by the group to develop its members’ capabilities. This sets group capabilities apart from individual capabilities, as the actions taken, as well as the functioning that is achieved, are collective.

Together, human capabilities, capability theory, and social identity theory offer a holistic lens for understanding family capabilities. Capability theory focuses on the real opportunities that families have to achieve well-being, while human capabilities refer to what families are actually able to do and be. Social identity theory adds insight into how family roles and behaviors are shaped by group membership and social expectations. Combined, these frameworks help to explain not only what families do, but also why and how they do it within their social and structural contexts. Yet, when human capabilities are examined through a family-centered lens, it becomes evident that these individual capabilities are insufficient. In addition, many group or collective capabilities underscore the significance of collaboration and purpose [42]. Collective capabilities are generated through an individual’s engagement in collective action, which entails what people do [43]. What families ‘do’, for example, processes, events, or rituals—things that have meaning for families at the moment of their occurrence—is important [44]. To understand family capabilities, a concept poorly defined in theory and the literature, examples of the choices that families make need to be explored, as well as how these choices are developed through the actions of family members [45]. The capabilities chosen by a family, as well as their priority, sequencing, and value, arise from family social realities, which, in turn, explicate the differences within, and between, families [46]. Consequently, an important conceptual divergence from standard accounts of the CA requires a move beyond merely exploring individual capabilities.

The research gap lies in the limited understanding of family capabilities. While the CA traditionally focuses on individual freedoms, this perspective is insufficient for capturing what families value, choose, and do together. Additionally, although collective capabilities emphasize collaboration, little is known about how these operate within families, i.e., how choices are shaped, negotiated, and expressed through family processes and priorities. Therefore, in this current study, the authors address this limitation by developing a new conceptual model to understand family through an ethic of care lens in two South African communities.

## 2. Materials and Methods

### 2.1. Study Design and Setting

In this qualitative study, the authors employed a Human-Centered Design (HCD) through a participatory action strategy, utilizing workshops and discussions with families, adolescents, and practitioners to gather and deductively interpret data. Human-Centered Design places people and their context at the center of research [47], while culturally specific methods are adopted [48] to communicate, interact, and empathize with, as well as stimulate, the people involved. In the South African context, to obtain an understanding of family needs, desires, and experiences, which often transcend those that the people themselves realize, HCD enables a communal approach and foregrounds the questions, insights, and activities of the families for whom the research outcomes are intended. This is both epistemological and ontological, since our positionality is to understand what families ‘do’ in terms of care, as well as comprehend how families produce care. In addition, it includes understanding that care values, as well as moral qualities and capacities, are important to families, and grow out of the process of care.

### 2.2. Location and Sampling

This research was conducted in partnership with families from two communities that experience enduring disadvantages, one urban community in Cape Town and one rural community in the Saldanha Bay Municipality. The sampling method was purposive, facilitated by the municipal government with the assistance of local agencies. This was important because of the trust developed between the families and the agencies through long-standing connectedness, as well as their embeddedness and daily relationships within these communities. In addition, this was essential for the management of risk for the families and the project team, particularly to overcome any suspicion of outsiders, as well as navigate the presence of gangs in the communities. The recruitment of participants was facilitated through purposive and snowball sampling, and subsequently, workshops were held at well-known community locations, including a community hall, an early childhood development setting, and a church. The authors initially interacted with family practitioners to gain access to families; however, the practitioners were also participants because of their embeddedness and roots in the local communities. Importantly, data gathered from the practitioners involved their own families, and not the families to whom they provided care. For this current study, the sample size was n = 53 (Saldanha Bay—4 practitioners, 12 adults, and 5 adolescents; Cape Town—8 practitioners, 15 adults, and 9 adolescents).

### 2.3. Data Collection Strategy and Analysis

**Figure 1:**
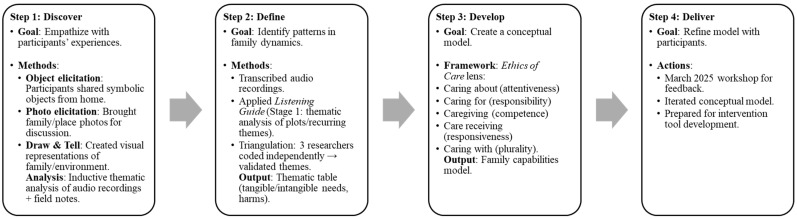
Steps in the data collection and analysis according to the HCD approach.

Data collection occurred in August 2024 and March 2025 by the means of a 3-day workshop that was hosted in each location. The two primary researchers, together with two team leaders, co-facilitated the workshops and discussions. Additionally, two research assistants and two research interns supported the process by managing audio recording, transcription, and taking detailed fieldwork notes. Each workshop lasted approximately five hours, including scheduled tea and lunch breaks. Following the initial data collection in 2024, a follow-up session was conducted in 2025 to verify the findings with the participants and gather further insights into family capabilities. The four steps of HCD, namely, discover, define, develop, and deliver, were drawn upon [49,50], ensuring methodological alignment with the participants’ lived experiences. Data were gathered through multimodal methods during the workshops, in recognition of the complexity, variety, and interrelatedness of families (Figure 1):

Step 1: Discover. This initial phase was centered on understanding and empathizing with the participants’ experiences. This approach builds trust, acknowledges participants’ needs, and finds possible solutions. In order to understand the participants’ family dynamics, various qualitative data collection methods were conducted. Firstly, the participants identified objects from their home environments that symbolized their family experiences. Object elicitation is a culturally specific and compassionate approach that enables participants to talk about their connectedness to an object, as well as its symbolic meaning and relatedness to family [51,52]. Secondly, the participants brought along photographs of their families and homes. Photo elicitation is also a culturally sensitive method that develops deep layers of meaning and contributes to the trustworthiness and rigor of research [53]. Finally, a draw and tell activity, where the participants created visual representations of their families, homes, and environment, added to the context and assisted in facilitating conversation and exploration with the adolescent participants [54]. Inductive thematic analysis was employed for this study, and facilitated the identification of patterns within and across data on the participants’ lived experiences, perspectives, and behaviors [55]. The data that were generated through these activities were audio-recorded with the participants’ consent, while detailed field notes were documented to ensure the integrity, trustworthiness, and rigor of the data collection process.

Step 2: Define. The second step of the process entailed generating an idea of what families do. To foreground the families, the audio-recordings generated from the workshops were transcribed verbatim and analyzed using the Listening Guide, a voice-centered, relational method of qualitative analysis that emphasizes the multiplicity of voices within a narrative and the social context in which they are embedded [56]. This method comprises the following four distinct stages: (1) listening for the plot and identifying repeated images, phrases, and dominant themes; (2) constructing “I-poems” to trace the speaker’s voice and sense of self; (3) listening for contrapuntal voices to surface internal tensions or multiple perspectives; and (4) situating the narrative within broader social, political, and cultural contexts [57]. For the purposes of this study, we focused exclusively on Stage 1 to inform the data analysis phase. Stage 1 involved a careful reading of the transcript to listen for the plot—what was happening in the narrative—and to identify recurring words, images, and patterns of meaning. In this study, Stage 1 was conducted through a thematic analysis, which involved familiarization with the data, systematic coding, organizing codes into patterns, and developing overarching themes that capture the shared experiences and perspectives within the data [57]. The thematic analysis was employed to generate common themes from the data, allowing for a structured interpretation of the participants’ narratives [58,59]. This approach enabled the identification of patterns and meanings across the data set, providing insight into shared experiences and perceptions. In particular, themes were developed which captured the personal and emotional dimensions of the participants’ reflections [60,61]. To ensure the reliability of the analysis procedures, multiple researchers were involved in the coding process. Specifically, three researchers independently coded the data, after which these themes were compared and validated across the broader data set to ensure coherence, depth, and analytical rigor. For validation, the method was presented to and read by academic peers, and initial ideas were presented to the participants in further workshops for further refinement and iteration.

Step 3: Develop. Based on the thematic analysis in Step 2, a conceptual model for the strengthening of family capabilities was developed through a process of care. Three broad themes, namely, tangible needs, intangible needs, and harms, were initially used to organize and merge sub-themes in a thematic table. Subsequently, these were interpreted through the lens of the ethics of care [62], specifically, to explore caring about (attentiveness), caring for (taking responsibility), caregiving (competence for care), care receiving (responsiveness), and caring with (plurality, how care is created and maintained). This approach explicates the interrelatedness of care in family dynamics among family members and is useful when exploring the boundaries, capacities, and capabilities of what families do.

Step 4: Deliver. In March 2025, the findings were delivered to the participants, and the authors engaged in further iteration of the conceptual model. This is an important step as it enables the refinement of thinking, utilizing participant experiences and understanding. In addition, it is the stepping-off point for the final step in the HCD process, not discussed here, as well as the focus of the authors’ ongoing work, namely, implementation, through the development of an intervention tool, to assist families.

### 2.4. Ethical Considerations

Ethics approval was granted by the University of the Western Cape (reference number: HS24/3/28). All participants provided their signed consent, with additional assent from parents or carers of adolescents. Before the session commenced, the participants were reminded that their participation in the study was voluntary and could be withdrawn at any stage, for whatever reason, without adverse repercussions. To ensure confidentiality and anonymity, the participants’ names were removed and replaced with pseudonyms.

Employing an ethics of care approach requires awareness of and sensitivity to the needs of others [63,64]. This is especially important in recognition of the difficulties that vulnerable populations in low-income South African families confront [11]. The authors aimed to develop an empathetic and flexible approach to ensure that it was meaningful and contextually appropriate. Additionally, they were observant of the post-pandemic rise in unemployment, food insecurity, and mental health difficulties [65] and responsive to the needs of the participants. It is important to note that the HCD approach was developed and adapted; therefore, the authors continuously reflected on their research methods, implementation, and findings. Additionally, they learned, alongside families, to understand what families ‘do’ in terms of care. Adopting this approach enabled the authors to focus on the needs and experiences of family life at the core. By understanding this, they were able to reconstruct what care ‘looks like’ in families.

## 3. Results

The first reading of the data, utilizing the Listening Guide, highlighted a variety of family structures, consistent with the existing literature, which the participants understood as the connections and relationships of family on the basis of tangible needs, intangible needs, and harms.

### 3.1. Tangible Needs

Tangible needs, such as medical assistance, housing, food, employment, and communication, are essential to ensure the well-being and stability of family members, providing a foundation for their physical, emotional, and social development [66,67,68]. For example, the participants disclosed the importance of family connections, as well as the fulfillment of their tangible needs. Despite financial challenges, some individuals expressed gratitude for even meager earnings, such as part-time jobs, which helped to provide basic necessities such as food, as follows:

*“You must be grateful for a spare job, maybe one of two days in the week that only pays R200, but even if you can buy bread with it, it’s fine, it’s food on the table.”*

*(Natalie, female, 38 years old)*

Parents and adolescents also valued shared activities, such as playing together or enjoying meaningful conversations, which strengthened their bonds and fostered human connections, as follows:

*“We play a lot together, he likes to run.”*

*(Sammy, female, 55 years old)*

*“I like the bond, I like the fact that we can share something in common and that we can speak about it and have a human connection. I speak with him any type of conversation, just speak about anything.”*

*(Chantelle, female, 27 years old)*

Additionally, there was a strong sense of communication within the families, with regular updates and messages exchanged to stay informed about each other’s well-being and events in the wider family network, as follows:

*“So, you get your message every morning and if anything happens within the other family units you get to know about it.”*

*(Amy, female, 50 years old)*

These actions reflect the roles of both material support and emotional connection in maintaining strong family ties. In addition, there was significant evidence of the role of women, including grandmothers, in caregiving, as well as meeting tangible needs, as follows:

*“In that drug house, she began giving birth. They called my other daughter to come and look because the baby is on her way… She phoned me to say that I [mommy] must now come. [I] mommy must come now! The child must go to the hospital…. This man rushed me to the hospital. When I got there, the baby was under pipes… The child survived.”*

*(Fiona, female, 58 years old)*

*“I wash him and give him his medicine. They didn’t want me to go with them to the hospital. They go with him but then they just drop the stuff by me. Like with the medication, I don’t actually know how to do it, but I give it. I read how to do it, then I give it to him. He can’t do anything for himself… He depends on me. I must I must provide for everything.”*

*(Lisa, female, 50 years old)*

### 3.2. Intangible Needs

Intangible needs, such as love, belonging, respect, compassion, connection, identity, laughter, and restoration, are important for emotional and psychological well-being within the family [69,70]. The concept of intangible needs focuses on aspects that are ‘felt’ (love), and, to some extent, taught (discipline) [66,67]. In the study, intangible needs were prominent, for example, “…trust, love, communication, understanding, loyalty.” (Aqeela, female, 43 years old). ‘Love’, or the lack thereof, emerged as a central theme, as follows:

*“Love is the most important thing.”*

*(Natalie, female, 38 years old)*

*“She loves me. Because she tells me.”*

*(Jack, adolescent boy)*

Others spoke of pride and affection for their family members, such as parents feeling proud of their children’s growth and achievements, even as they became more independent, as follows:

*“I’m very proud of him, he’s working now. He has a girlfriend and sometimes he doesn’t need his mommy anymore. His mommy is very proud of him.”*

*(Saskia, female, 50 years old)*

Respect and discipline were also highlighted as key values within families, often intertwined with love, as well as the understanding that these qualities form the bedrock of healthy relationships, as follows:

*“You must respect your mother and father so that you can respect.”*

*(Sara, female, 40 years old)*

*“The respect, the discipline. It’s not about material things, material stuff. It’s always about respect and discipline and love. The love they give to you, they share that love. You don’t give that, you leave it there.”*

*(Frank, male, 53 years old)*

Overall, the emphasis was on the deep connection of love, respect, and discipline, with family members sharing affection and valuing mutual respect as essential components of their bonds. Additionally, intangible needs crucially facilitated separation from long-standing trauma. Joe (male, 47 years old), for example, discussed his history of harm, such as being burned as a child and removed into foster care and then a safe house as an adolescent, as well as difficulties with relationships. However, a meeting with his father in adulthood, to understand his childhood, enabled him to begin to rebuild trust and self-esteem, maintain relationships, and “break the chain” of harm.

### 3.3. Harms

Harms are multifaceted, and include loss, poor health, accidents, violence, abuse, drug and alcohol misuse, and wider social ills, such as poverty, misogyny, and gangsterism [71]. Joe was aware of the harm to himself and, by extension, those around him, “I used to smoke drugs… I couldn’t face myself.” (Joe, male, 47 years old). This acknowledgment of not coping is also an acknowledgment of caring for oneself in a particular way and not caring about others [72,73]. Harm, therefore, is also experienced when care within the family is ‘bad’ [74,75,76]. Bad care occurs, for example, through abusing and abusive behavior, when caring responsibilities are not shared equally, including when the primary caregivers have unmet care needs of their own or when poor communication and a lack of trust are evident [74,77,78].

*“… a home is not always a safe space for everybody to be around because if you’ve been raised by abusive or been abused then that home is not going to be a safe space.”*

*(Sasha, female, 55 years old)*

*“Since my mother passed away, I never really had someone to speak to… Because sometimes families, they don’t have time to listen to what you, you know, to you. Or they have their own problems and whatever they have. So, they don’t want to listen to you…Even though I’m not working… I do whatever I… I give you whatever… But it’s never enough. Whatever I do is never enough.”*

*(Fadwa, female, 40 years old)*

Life experiences were often described as taxing, propelling individuals into a state of anxiety and reluctance to share their apprehension for fear of being judged or uncertainty about the source of appropriate support, as follows:

*“I also lost my daughter seven years ago to sudden infant death syndrome…. I don’t have a mommy or daddy anymore, and since they died the rest of my family wrote me off.”*

*(Alana, female, 50 years old)*

*“Life experiences can be very taxing, very difficult. It pushes you to a place where you do not really want to share because you feel people are going to judge you.”*

*(Eve, female, 52 years old)*

Consequently, help-seeking behavior was limited among many of the participants, who envisaged their only way out of harm as being through a much higher authority, as follows:

*“Only God [helps me]. That’s the only person… To God I can talk to. And that is confidential. I can talk to God. He made me strong and He made me what I am [he’s the only one I trust]… I can’t go to my sisters or my brother.”*

*(Eve, female, 52 years old)*

Further analysis of the data expanded to examine broader networks of care, highlighting the dynamic and ongoing processes of caregiving within the family and how these relationships are negotiated, sustained, and transformed over time.

### 3.4. Caring About

‘Caring about’ focuses on acknowledging and being attentive to the needs of the family [79,80]. The authors’ analysis revealed that attention was placed, universally, on fundamental necessities, such as access to food, appropriate clothing, and stable housing, highlighting the importance of financial security, as well as education and employment, to provide a better quality of life for children and the family. Beyond these tangible needs, emotional support and guidance emerged as essential, with attention focused on a loving, nurturing environment, a sense of safety, and strong, meaningful relationships, as follows:

*“There’s food…suitable clothes for them.”*

*(Natalie, female, 38 years old)*

*“The father left and we don’t worry anymore, so I did promise them a better life than I have. I did not do matric, and I just want to work… At this moment, that is all that matters to me now, to give them a better life than I have… I’m looking now for work to give them a better life and for us to live together in our own house, alone without the father.”*

*(Lucy, female, 20 years old)*

Although these needs were not always met, the participants were attentive to these needs, and consequently, were also attentive to the harms arising from unmet tangible and/or intangible needs, as well as the ‘caring deficit’ [81] (p. 332), denoting insufficient attention paid to needs across the family, as follows:

*“My real mother kept me in school but my father took me out. So, I don’t feel like going anymore. My stepmother hasn’t worked for three or four years now… She just scolds”*

*(Bobby, adolescent boy)*

*“… I was with my mom and dad. When I was one, my father went to Potchefstroom and my mom stayed here. I haven’t seen him for seven years… He doesn’t make time for me anymore, he doesn’t come to me. When I was four years old, I got a stepdad, a policeman. That guy was a mad head. Some evenings he would then chase me, he wants to shoot me in the house.”*

*(Ashley, adolescent girl)*

### 3.5. Caring for

The notion of ‘caring for’ within the family focuses on the individuals who hold the responsibility of providing care to family members once their own need(s) have been met. Remarkably, those who assumed or were delegated the responsibility of caring for their families were predominantly females, namely, mothers, grandmothers, and female children. These women realized the need to seek employment opportunities, have and maintain a relationship with their children, be the protector of the family unit, and be financially stable, as follows:

*“Sometimes they communicate with their father, but they don’t have a bond so they just have a relationship with me.”*

*(Lucy, female, 20 years old)*

*“Me as a mother, [I] need to fulfill the responsibility as a mother… I’m bringing the protection of the Lord always, I keep it always. I am the protection of the Lord for my family… I must be on my knees for my family… I need to ask, Lord, protect my children. I am the covenant…as the protector of my family.”*

*(Vivian, female, 51 years old)*

These data also reveal an intergenerational transfer of care, where the responsibility of caring for family members is handed down from one female primary caregiver to another, reflecting both cultural expectations and deeply embedded family norms that shape how care is understood and enacted over time, as follows:

*“…the one thing that Ma [grandmother] did was before she passed away, she said, you need to get the family together. But I never. I didn’t want to do that. Why should I be doing it? She had kids to do that.”*

*(Danielle, female, 36 years old)*

*“My mother is the head of the household now. She took the lead after my grandmother when she was small and now it’s me. It’s the intergenerational expectations.”*

*(Alice, female, 40 years old)*

### 3.6. Caregiving

‘Caregiving’ requires competence, as well as specific skills, and involves physical, emotional, and spiritual support in daily life. Additionally, caregiving includes providing practical assistance and personal care [80,82], as follows:

*“I had to give a little push to finish. I had to get some substance just to uplift them, she’s got exams, keep her mind awake especially the stuff that she forgets.”*

*(Joe, male, 47 years old)*

*“…[family] they do the washing and hang clothes a lot [by my mother and grandma]. My ma, she does the washing during the week and if she comes late to the house then she can only do it on the weekends. She’s only off on Sundays.”*

*(Justin, adolescent boy)*

*“I must make food for them [babies]. I must take them out, let them play in the park. I must put some of them to sleep.”*

*(Jessica, adolescent girl)*

Caregiving also encompasses emotional and spiritual support, including practices such as offering prayers for the safety, health, and overall well-being of loved ones, which reflect deep relational ties and the moral responsibilities that families assume for one another [83,84], as follows:

*“I need to be on my knees for my family… I need to be in contact with the Lord to pray for my children, for their safety and their well-being for my family.”*

*(Vivian, female, 51 years old)*

However, caregiving could be perceived as ‘bad care’ when caregivers consider the role as an aspect of the colloquial ‘black tax’ [85,86]. This term is increasingly used in South Africa to describe the obligation placed upon successful young Black people to financially support and care for their extended family, reflecting deep-rooted cultural expectations shaped by historical inequality and limited social welfare support [85,86], as follows:

*“It’s toxic. Love is conditional and unaffectionate. It’s the black tax. I must give money to my mother… so it’s like a psychological binding or conditions for affection.”*

*(Chloe, female, 29 years old)*

*“[Family] They see me with money. Miss money bags. With money bags. Because I always have and they always expect me to have. Sometimes I don’t have then they think I’m lying. But that is what they see me as. Here she comes, she’s got money.”*

*(Saskia, female, 50 years old)*

Additionally, caregiving could lead to ‘bad care’ when intangible skills, or the moral competence for care, are absent [87,88], as follows:

*“My family…I just think of all the bad things I did to them. I feel like I never did something good in their eyes. They see me as a nuisance. We just argue. So, they refuse to help you. That’s why I never ask for help. Because you get left out.”*

*(Basil, male, 46 years old)*

### 3.7. Care Receiving

The act of ‘care receiving’ is a reciprocal relationship within families. To be able to receive care, family members need to reflect on their caregiving and be open to receiving care in return [78,80]. This reflective process creates a balance in the care process, where individual not only provide support, but also acknowledge the value of accepting help, when needed, as follows:

*“I’m going to need that safe space. So, I will continuously look out for my safe space whenever I’m in need…let’s say for money or for a nice time or for food. That must be my safe space…I think regardless of gender, it’s the person that you feel from the go ahead, you established that relationship and that person made you feel wow. I hear you, I see you. I can support you in any life decisions that you make, whether it be good or bad, because even if it’s bad, that person can still steer you in the right direction.”*

*(Eve, female, 52 years old)*

This reciprocal relation involves reason (being attentive, recognizing care needs, and taking responsibility for care and caregiving) and emotion, and these are in constant relation [89].

*“I will tell them, everyone close to me that makes me feel safe, that I want to make them feel safe, my children, mother, father. I will tell them like this, everyone who’s got respect for me, I will call ‘family’.”*

*(Natalie, female, 38 years old)*

However, there are many examples where care receiving is an aspect of ‘bad care’, because caregivers are reluctant to request care. For example, Eve, a 52-year-old female, when asked what she implied when she said, “I can’t be everything, to everybody, all the time”, disclosed her own care needs, as well as how they remained unmet. Subsequently, when asked whether she had requested assistance, she replied in the negative, because it was important for her to remain strong. This sentiment was also expressed by Vivian as needing “to be on her knees for her family all the time”. In addition, she added that, within her culture, she was not expected to ask for help for fear of appearing weak.

Care receivers’ hesitation to articulate their needs emerges most prominently in familial contexts characterized by eroded trust, where historical patterns of unmet expectations or relational instability have created psychological barriers to vulnerability and help-seeking behavior, as follows:

*“They take it [the need to receive care] somewhere else or their behavior becomes disruptive, then they look for other people, to like confide with, or even trust. That’s where their behavior escalates to addiction.”*

*(Danielle, female, 36 years old)*

This lack of reciprocity and a disjuncture between reason and emotion in caregiving and care receiving could also lead to animosity and anger [90,91]. For example, Sarah, a 42-year-old female, disclosed that she would not request assistance or disclose anything to anyone in her family for fear of rumors and gossip being spread, for which, “I become a little bit revengeful when it comes to others, which is not the way to go. It’s not supposed to be that way. But when I would give you an answer by telling you blindly no, it would be because you have done something to me, or you have given me the same attitude once upon a time.”

### 3.8. Caring with

The notion of ‘caring with’ is a constant act of care within the family domain; however, caring with may only continue through a continuous cycle of reciprocity, communication, trust, discipline, respect, and love [92,93]. There was evidence of spending time together, especially from the young participants, as follows:

*“We’ll watch a movie or study with my parents.”*

*(Jack, adolescent boy)*

*“We go swim. My family. We swim. We play in the sand. We eat.”*

*(Kyle, adolescent boy)*

*“We like to watch movies and stuff or like play games or like just listen to gospel and sing along.”*

*(Justin, adolescent boy)*

However, the older participants also understood the importance of respect and care within their families, and there was evidence of parents teaching children to respect their home, as well as reciprocity, as follows:

*“My mother loves God and she knows and teaches us the rules, what to do and what not to do. I grow them up by myself and they are very understanding of each other. I was sick and then my eldest son was very concerned about me, that I must eat.”*

*(Maddie, female, 57 years old)*

Additionally, certain families demonstrated an adaptive approach to collective well-being by intentionally cultivating democratic family structures, wherein shared decision making, mutual respect, and equitable participation in household governance became foundational to their daily functioning and relational dynamics, as follows:

*“When we got out as a family together and just share our perspectives and our experiences and just be connected with each other on a human level…It is so important to spend time with each other because we don’t know what tomorrow holds…It is nice to know that we share a same bond where we can say that this is my family, this is who I relate to.”*

*(Chantelle, female, 27 years old)*

The ethics of care transcend the meeting of immediate needs to foster empowerment and adaptability among individuals and families [94]. The focus on care helps to explore what families do, specifically, their capacities and approach to family well-being. A care-orientated approach offers a way of fostering a more equitable society and is complementary to the aims of the CA [2,3]. Both approaches are interested in relationality (with wider social structures as well), are justice-oriented, and seek to address injustice through empowerment [95]. Relations in the CA have intrinsic ethical value [96]; therefore, family capabilities involve the moral capacity, as well as other abilities, to care within an intrinsic ethical frame. A summary of the main themes, along with their corresponding findings, is visually presented (Table 1).

**Figure 2:**
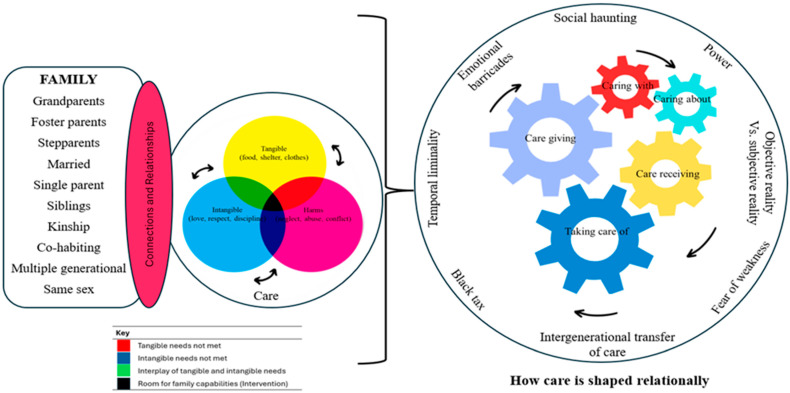
A care-centered model for family capabilities.

Building on these findings, the authors propose a novel care-centered model for family capabilities (Figure 2) that systematically integrates the following eight key themes emerging from the data: tangible needs, intangible needs, harms, caring about, caring for, caregiving, care receiving, and caring with. The model’s development was guided by a rigorous HCD process, ensuring methodological alignment with participants’ lived experiences. Utilizing the HCD approach assisted in portraying each thematic element within the model in an authentic manner that reflected the realities experienced by families while collectively addressing the complex dynamics of family capabilities.

## 4. Discussion—A Care-Centered Model for Family Capabilities

The model focuses on the intertwined relationship between diverse family structures and care practices, encompassing both tangible and intangible aspects of care, as well as the potential consequences of harm. Tronto’s [62] ethics of care process has been integrated to provide a contextual framework, and the model includes examples of harms that impede family capabilities, namely, those factors that interrupt or deny opportunities to achieve the ‘beings and doings’ required for well-being.

### 4.1. Family Structures

Different family structures, such as grandparents, foster parents, stepfamilies, married parents, single parents, and same-sex families, influence the way that capabilities are achieved within a family. A family, as a group of people that has congregated to care for and support its members, utilizes resources and engages in practices, processes, and arrangements for family members to achieve well-being. Family members can be proximal or apart; however, they should rely on each other for support. In family capabilities, as with collective capabilities, well-being is enhanced when the family is supportive and engages in an ethical process of care [43,97]. However, each structure presents unique challenges and strengths when meeting the needs of its members. For instance, single-parent families may experience greater financial and emotional strain, potentially affecting the provision of both tangible and intangible care. Similarly, for some, the mother or grandmother may be perceived as the ‘glue’ that holds the family together. Therefore, when that ‘glue’ is nonexistent, assuming the role of maintaining family cohesion becomes a challenging task for another family member. Consequently, a void develops within the family dynamic, which ultimately causes families to disintegrate or become disconnected.

In contrast, multigenerational families could offer stronger emotional support networks, but may face challenges with decision making and household role allocation [98,99]. The presence of siblings and kinship ties could also impact caregiving responsibilities, with older siblings often stepping into parental roles when required. A family’s unique approach to ‘doing and being’ within its structure (the nature of relationships and familial connections) ultimately shapes how tangible and intangible needs are met, as well as how pathways for ‘harms’ and setbacks to capabilities are developed.

### 4.2. Tangible and Intangible Care and the Consequences of Harms

Need within families can be categorized into tangible and intangible forms, both of which are crucial in order to understand well-being. Tangible needs include the provision of basic necessities, such as food, shelter, and clothing, which are essential for physical survival and security [100]. Intangible needs include love, respect, dignity, and discipline, which foster psychological well-being and a sense of belonging [100,101,102]. Meeting both tangible and intangible needs is necessary for holistic family well-being; however, when these care needs are not met, families may experience various forms of harm. A lack of tangible care could result in poverty, homelessness, and physical health issues, while insufficient intangible care may lead to loneliness, emotional distress, low self-esteem, and strained relationships [102]. A parent who works away from the family home may do so to provide the financial means to meet tangible needs; however, their absence could lead to inequitable caregiving roles for others in the family, as well as loneliness and a sense of loss.

Tangible and intangible needs are also entwined. Family members acknowledge the feelings of love within their family, which is often associated with acts of giving and sharing, to meet tangible needs. For many care receivers, this is what families are all about, ensuring that they are cared for and their well-being needs are met. Our results are similar to another study where it was reported that acts of love help to maintain a sense of belonging to the family [97] while providing the basic necessities necessary for well-being. However, for some caregivers, these acts (the expectation of giving and sharing) may be a tax within the family, being indicative of ‘bad’ care and constraining family capabilities. Consequently, addressing tangible and intangible needs and understanding their interplay in mediating or enabling harm are vital to family capabilities.

In Figure 1, the interrelated elements of the process are shown as different sizes to illustrate that family capabilities are not equally experienced across each element, and the size of each element (representing the opportunity to achieve well-being) is unique to each family in relation to the social, structural, and other harms experienced within the family. Historical injustices and socioeconomic disparities, especially those resulting from apartheid-era policies, continue to affect the family relations and vulnerabilities of South Africans living in both urban and rural areas [103]. Similarly to previous studies, social haunting (modes of being and doing associated with historical injustices) [104] and temporal liminality (how families continue to transition between apartheid and post-apartheid eras) affect moral capacity and are compounded by systemic inequalities in education, employment, and healthcare, which perpetuate cycles of poverty and social exclusion, disproportionately impacting Black and working-class families [105].

### 4.3. How Care Is Shaped Relationally

The CA and ethics of care, in contrast to universal ethical theories, emphasize the contextual elements of moral judgements [89,106]. Our model is aligned with previous research where it was indicated that relationality is part of the concept of capabilities, where relations have intrinsic ethical value [96]. Therefore, family capabilities involve the moral capacity, as well as other abilities, to care within an intrinsic ethical frame. In their approach, the authors explored moral capacity and action through a framework of five elements, namely, attentiveness, responsibility, competence, responsiveness, and caring with [62,107]. Similar to our study, these elements correspond to Tronto’s [62] process of care, involving caring about, caring for, caregiving, care receiving, and caring with, each shaping families’ capabilities, namely, their ‘beings and doings’.

### 4.4. Caring About

Family capabilities involve being attentive to the needs of others, implying that when individuals are not attentive to the needs of others, it becomes impossible to address those needs and fulfill the opportunity for well-being [108,109]. In alignment with another study, attentiveness emerged as a critical component of families’ ‘beings and doings’ through both emotional sensitivity and tangible actions, such as ensuring the physical and emotional well-being of their loved ones [109]. This attentiveness fostered emotional security and strengthened family bonds, reinforcing the role of care in maintaining cohesion. However, from our study, the absence of attentiveness had adverse effects, leading to neglected emotional or practical needs. Requesting assistance among family members was often met with dismissal, reinforcing feelings of isolation and distress, as well as a lack of help-seeking behavior [110]. This lack of attention not only prolonged their emotional challenges, but also contributed to maladaptive coping mechanisms, further exacerbating social disconnection [110].

### 4.5. Caring for

Family capabilities also involve the manner in which the responsibility for an identified need is assumed, as well as how the response to that need is determined [46,111]. Similar to previous authors, responsibility for care was primarily gendered, with women assuming the central caregiving role within families [111,112,113,114]. This expectation was deeply embedded in cultural traditions, with mothers and grandmothers expected to carry the emotional and physical labor of maintaining family stability, specifically, to be the ‘glue’ that binds the family [113]. The persistence of these gendered norms underscores how family capabilities are shaped by inherited structures, often reinforcing unequal distributions of care. While some shifts in the acceptance of responsibility were noted, they remained subtle, and the burden continued to predominantly fall on women [112,113]. These results are consistent with findings that mothers and grandmothers are the primary carers in the South African context [112,113]. The responsibility for grandchildren, taken or given to grandmothers, was also proximal, especially when the mother or father worked outside the home or was otherwise unavailable. The grandmother, therefore, was perceived as having time to offer care [114,115]. The freedom of the person assuming the role of the ‘glue’ within the family to make effective choices and choose from a range of available options is limited. This suggests a need to reimagine a more equitable distribution of responsibilities, as well as strengthen overall family capabilities, by promoting shared caregiving efforts, especially when the death or absence of the ‘glue’ results in a care void and subsequent family dysfunction.

### 4.6. Caregiving

Caregiving within families requires practical skills, as well as moral competence, emphasizing the importance of learned behaviors and ethical responsibility in the provision of support. The participants highlighted that caregiving involved more than the mere execution of tasks, as it also included the demonstration of empathy, patience, and ethical commitment. While the participants frequently relied on moral competence, as well as faith, to navigate their caregiving roles, Bozalek et al. [116] and Tronto [62] argue that effective caregiving depends on access to adequate resources, including time, financial support, and essential skills, including the development of moral competence. Thus, from our study, the intergenerational transmission of caregiving skills reinforced cultural expectations, particularly for women, while faith emerged as a significant factor in the shaping of moral competence in care [112,113].

Faith has previously been reported as an emotional coping mechanism for families [117,118,119]. Our study aligns with the findings of Hooyman and Kramer [117], which reveal that while faith may provide emotional endurance [118], structured external support is essential to prevent burnout, as well as alleviate the disproportionate burden on women [119]. However, in some instances, faith was also the only form of care that caregivers received. DuBose [120] describes faith as an existential coping mechanism that helps caregivers to find meaning amid exhaustion and uncertainty. Many participants disclosed that religious beliefs guided their sense of duty and perseverance and allowed them to navigate caregiving roles with greater emotional resilience. However, previous research argues that when faith is the only source of support, concerns about its sustainability emerge. Ng et al. [121] argue that while faith can offer emotional strength, it cannot replace the essential structural support which caregivers need. Additionally, Xu et al. [122] assert that, while faith provides resilience, it cannot prevent the burden on caregivers without proper systemic support.

This focus on faith also illustrates that a collective and equitable understanding of and approach to caregiving within families could be missing [122]. Family capabilities acknowledge that people are a part of familial and other social structures, and what families accomplish together shapes their chances and successes. For example, in a family where unemployment and poverty are prevalent, members could develop an approach that works to improve the chances for everyone. They could be attentive to the needs of all and take an equitable approach to assuming responsibility for and responding to needs by combining their money, skills, and knowledge to form a collective strategy that faces economic problems which affect their well-being. Robeyns [40] and Rosignoli [123] assert that a group or collective must participate in joint action to reach a capacity that the group’s members value. Therefore, a family’s collective capabilities are those that the family uses to develop its members’ capabilities, including acknowledging that caregivers also have needs. This sets family capabilities apart from individual capabilities, because the actions taken, as well as the outcomes achieved, are collective and aspects of shared moral competence.

### 4.7. Care Receiving

Care receiving and responsiveness to care underscore the importance of reciprocity in family ‘being and doing’. Authors have highlighted how women fulfil a greater role as family caregivers [124,125], as well as how gender plays a significant role in both the provision and receipt of care [126]. However, the care needs of family caregivers are frequently neglected, despite the complexities inherent in their caregiving responsibilities [125]. Women spend most of their time providing care without any help or support [124], a care imbalance that could lead to emotional exhaustion and neglect, strain, burnout, depression, and reduced well-being [125,127]. Reciprocity of care encompasses not only financial and physical exchanges, but also the expression of care, which includes in-kind and economic support, as well as social, emotional, and psychosocial support [126].

Family capabilities can be strengthened by fostering greater reciprocity, ensuring that care is not only given, but also acknowledged and returned. Nussbaum’s understanding of capabilities reiterates that society functions as a caregiving and care-receiving entity [128]. Consequently, family capabilities are augmented through the reciprocal nature of care, facilitated by the capabilities of affiliation and emotional connection. Without this balance, caregivers face heightened emotional and physical consequences, which ultimately weakens the family’s ability to function as a supportive unit. Therefore, this study found that a caregiver’s autonomy within the family, her ideal sense of her ‘self’, is affected by the impact of familial and social structures, as well as harms. This ‘relational autonomy’ [129] acknowledges the bifurcated nature of caregiving, as well as care receiving, suggesting that family capabilities should be attentive of power while seeking to understand how to change the silencing of a caregiver’s care needs, instead of reinforcing the expectation that caregivers should adapt to their circumstances, privately, by demonstrating resilience [129].

### 4.8. Caring with

Family capabilities are essentially built on the moral capacity and ability to care for people, in a context where relationships have intrinsic ethical value. This is aligned with Held [89] (p. 10), who argues that the “focus of the ethics of care is on the compelling moral salience of attending to and meeting the needs of others for whom we take responsibility”. Care involves emotion, as well as reason, and the reciprocal nature of caring with one another within the familial context is built on trust [130]. The findings from this study suggest that caring with includes the opportunity to share activities that foster emotional connection and mutual support. The adolescents in this current study identified shared experiences, namely watching movies, playing games, or engaging in family meals, as meaningful expressions of care. Similar to previous research, these activities provided a space for emotional bonding, moral attunement, and validation, reinforcing the importance of relational care within families [126].

Trust enables individuals to acknowledge their ability to rely on one another in caregiving and care-related activities [131,132], which may explain why many adolescents in this current study highlighted the importance of trust and caring within their families [133]. However, the parents did not always articulate these moments in the same way, often prioritizing their roles in meeting tangible needs over engaging in reciprocal emotional connections and meeting intangible needs. Not only does this difference in perception between adolescents and parents suggest the need to acknowledge and nurture shared experiences as a vital component of family capabilities, but it also highlights that the capabilities chosen by a family (their priority, sequencing, and value, arising from family social realities) are not linear.

To understand the capabilities chosen in a family (the combinations of potential functionings that they value and access) to achieve well-being, individual consciousness and collective action need to be understood. The approach must include a process that captures how families think, decide, and do, as well as the interplay between individuals, collectives, resources, and historical and material experiences. This implies that the ‘collective capabilities’ developed among the family unit, as well as the ‘collectively dependent personal capabilities’ developed by individuals within the family unit, must be justified ([40] p. 116, [43]). In this context, family capabilities that are based only on personal, social, and environmental circumstances are lacking, as this conceptualization fails to capture ‘action’ (how the consciousness of self, family, and the material conditions of family lead to action for collective and individual well-being). Caring with, in this context, is a ‘point d’appui’, or leverage, to understand what families actually do to achieve well-being.

Families function within a framework of care that is always present, though its quality varies between ‘good’ and ‘bad’ care [62]. ‘Good’ care does not only address immediate needs [14,134], but also involves caring with and creating opportunities for well-being that balance resources, selfless motivations, reciprocal relationships, and long-term family stability [135]. ‘Bad’ care does not indicate an absence of care, but rather inadequate or neglectful care, resulting from systemic barriers, socioeconomic constraints, or fractured family dynamics [89], such as emotional barricades, fear of being perceived as weak, or incapacity in help-seeking behavior. Our study aligns with previous research where it was reported that this instability limits a family’s ability to offer the attentiveness and competence required for caregiving [136,137].

When care is insufficient, or ‘bad’, it results in various disturbances that affect the stability and well-being of family members [89]. This disparity between objective reality (the actual conditions of the family) and the subjective ideal (individual perceptions and experiences of ideal family capabilities) could cause a silencing and misrepresentation of care needs, and consequently, a loss of opportunity in accessing capabilities, as well as a fractured approach to ‘caring with’. Similar to previous authors, this disparity is often exacerbated by systemic socio-inequalities and gaps in social policies that fail to adequately support families’ diverse needs. Such structural limitations can deepen the experience of ‘bad care’ by restricting access to resources and undermining collective caregiving efforts [74,75,76]. Thus, improving the capacity and responsiveness of local social services is crucial to addressing instances of ‘bad care’ and ensuring that families receive the support necessary to fulfill their caregiving roles effectively.

### 4.9. Practical Application of the Model

The proposed care-centered model for family capabilities offers actionable pathways for state and local social services to enhance family support systems. By addressing tangible needs, relevant and context-specific programs could integrate material assistance (e.g., housing subsidies and job training) with intangible needs (e.g., family counseling and parenting workshops) to foster holistic stability. Social workers (or equivalents, such as Child and Youth care workers and Departments of Social Development) could use the model’s harms component to identify at-risk families and provide trauma-informed interventions, while its caring about dimension could guide needs assessments to tailor resources. The caring for and caregiving elements could inform training for caregivers, particularly women, who disproportionately carry these roles, with respite care or skill-building programs. To address barriers to care receiving, services could design peer-support networks that normalize help-seeking behavior, and the caring with principle could shape community-based initiatives to strengthen collective bonds. By embedding these themes into policy frameworks, the model could transform service delivery from crisis management to capability building, aligning with broader goals of social cohesion.

### 4.10. Strengths and Limitations

The key strength of this current study lies in the participatory and context-specific approach, which involves direct participation, as well as engagement with families, adolescents, and practitioners. By employing a HCD, the authors ensured that the developed conceptual model is grounded in lived experiences, enhancing its applicability and relevance within the South African context. Additionally, the use of the Listening Guide approach, alongside thematic analysis, provided a rich and layered understanding of family capabilities, capturing both individual narratives and broader patterns in family dynamics. However, this study has certain limitations that need to be addressed. The sample was drawn from only two locations, Saldanha Bay and Cape Town, which may limit the generalizability of the findings to other regions in South Africa. Future studies, therefore, should consider involving multiple regions across South Africa to enhance the generalizability of the findings and strengthen the understanding of family capabilities across diverse sociocultural and economic households. While the study incorporated diverse perspectives from practitioners, adults, and adolescents, future research could benefit from a larger, more geographically diverse sample to further validate the model. In addition to the geographic limitation, the use of purposive and snowball sampling may have resulted in a sample that is not fully representative of the broader population. Future research could address this limitation by incorporating more diverse recruitment strategies, including stratified or random sampling across multiple regions, to enhance the applicability of the findings. Additionally, the reliance on qualitative data suggests that the findings are subjective in nature and may not capture the full complexity of family capabilities in a measurable way. Therefore, future studies, aspiring to explore similar initiatives, could integrate mixed-methodological approaches to strengthen the empirical robustness of the model.

## 5. Conclusions

The findings of this current study contribute to the understanding of family capabilities by conceptualizing a model that draws on a relational process of care, embedded within families’ everyday ‘being and doing’. The CA centers on an individual’s genuine opportunity to live a life they value [25,138]. Its significance lies in moving beyond subjective well-being or access to resources to a focus on actual ‘beings and doings’, such as being in good health and having access to valued opportunities. The concept of family capabilities, building on the CA and collective capabilities, focuses on the relational nature of family life within a moral frame. To support this, the authors drew on Tronto’s [62] ethic of care, which aligns with the CA through its emphasis on justice, relational interdependence, and context-specific responses to human needs [139]. In this study, the authors highlight the centrality of care in shaping family capabilities, positioning the family as the foundation and outcome of care. The proposed model offers a relational understanding of how families navigate care through both tangible and intangible means, while acknowledging the potential harms that may arise. By embedding care within social, cultural, and temporal contexts, the model reveals how family capabilities are developed, sustained, or constrained. It also draws attention to the reciprocity of care, as well as the often-overlooked needs of caregivers. The model is grounded in Tronto’s five care processes, namely, caring about, caring for, caregiving, care receiving, and caring with, which raise important questions around attentiveness, responsibility, competence, and responsiveness. This perspective has implications for policy and practice, calling for support systems that strengthen families, promote equitable caregiving, and foster holistic well-being. It is intended for use by practitioners, policymakers, and community-based organizations seeking to design or evaluate family-centered support interventions. By highlighting how families actively shape care in their own contexts, the model offers a practical and conceptual tool to guide more responsive, relational approaches to care.

## Figures and Tables

**Table 1:** Main themes and their corresponding key findings.

Main Theme	Key Findings	Notable Results
1. Tangible Needs	Essential for physical/emotional stability (housing, food, jobs, communication).	Even minimal income (e.g., part-time wages) was critical for basic needs.
		Women/grandmothers dominated caregiving roles, including medical and financial support.
2. Intangible Needs	Love, respect, and discipline foundational for emotional well-being.	Love was universally emphasized as central to family bonds.
		Trauma healing occurred through reconnection (e.g., repairing broken relationships).
3. Harms	Multifaceted: abuse, poverty, loss, and “bad care” (unequal responsibilities).	Homes often unsafe due to abuse or neglect.
		Participants relied on faith over family for support due to distrust.
4. Caring About	Attentiveness to needs (financial, emotional, safety).	Mothers prioritized children’s education/future stability.
		Tensions arose from intergenerational care obligations (“black tax”).
5. Caring For	Primarily shouldered by women (mothers/grandmothers).	Care roles passed intergenerationally (e.g., daughters assuming caregiving).
		Spiritual care seen as protective (e.g., prayer).
6. Caregiving	Required competence (practical + emotional/spiritual support).	“Bad care” resulted from lack of affection/skills or resented obligations.
		Adolescents often provided childcare/household support.
7. Care Receiving	Reciprocity hindered by pride, distrust, or cultural norms.	Caregivers avoided requesting help to avoid perceived weakness.
		Lack of trust led to isolation or externalized coping (e.g., addiction).
8. Caring With	Sustained through reciprocity, respect, and shared activities.	Adolescents valued shared activities (e.g., movies, swimming).
		Democratic family dynamics fostered mutual respect.

## Data Availability

Data is unavailable due to privacy and ethical restrictions.

## Abbreviations

The following abbreviations are used in this manuscript:

CA	Capabilities Approach
HCD	Human-Centered Design
NDP	National Development Plan
SIT	Social Identity Theory
